# Microbiome-guided rhizosphere engineering for sustainable remediation of agricultural soil contaminants

**DOI:** 10.3389/fmicb.2026.1994433

**Published:** 2026-10-28

**Authors:** Priya Arunkumar

**Affiliations:** Division of Biotechnology, Karunya Institute of Technology and Sciences, Coimbatore, India

**Keywords:** bioremediation, microbiome-guided remediation, rhizosphere engineering, soil contamination, sustainable agriculture

## Abstract

Agricultural soils increasingly carry mixtures of pesticide residues, heavy metals, petroleum hydrocarbons and microplastics, and conventional phytoremediation is too slow and too contaminant-specific to address them. Rhizosphere engineering, the deliberate manipulation of root-associated microbial communities, is widely proposed as the alternative, but the literature reports laboratory, pot and field results in a single register, so a reader cannot easily tell which approaches are deployable. This mini-review stratifies that evidence rather than surveying it. Every approach examined is assigned to one of three tiers: field-validated, emerging at pot or microcosm scale, or conceptual. Only two domains prove to be field-validated, namely metal immobilization and phytoextraction through bioaugmentation and bulk amendment, and hydrocarbon rhizoremediation by defined consortia; both rest on cultured isolates rather than engineered organisms. Microplastic rhizoremediation has not been demonstrated in soil, and we separate interception, adsorption, weathering, transformation and mineralization to show why reported removal is usually not degradation. Synthetic biology, CRISPR-based community editing and computational design remain conceptual, and we set out the horizontal gene transfer, containment, non-target and regulatory obstacles that stand between them and field use. We identify the measurements that would move an approach from one tier to the next, and argue that the principal deficit in this field is not technical capability but field-scale evidence reported to a common standard.

## 1 Introduction

Agricultural soils are accumulating an increasingly complex burden of contaminants. Pesticide residues, heavy metals and metalloids, petroleum hydrocarbons and, more recently, microplastics and their associated additives now co-occur in many cropping systems, delivered by agrochemical application, irrigation with contaminated water, sewage sludge and biosolid amendment, atmospheric deposition and the use of plastic mulch films. These contaminants rarely occur singly, and their combined presence creates toxicological and remediation problems that cannot be resolved by treating one pollutant class at a time, because competition for sorption sites, shared detoxification pathways and carrier effects make the behavior of a mixture different from the sum of its components (Rai et al., 2023; Zhai et al., 2024; Kundu, 2026).

Conventional phytoremediation addresses this burden only partially. Some plant species can sequester or degrade soil contaminants, but the approach is constrained in three respects. It is slow, commonly requiring several growing seasons before a measurable reduction is achieved. The hyperaccumulating species on which metal extraction depends typically combine a narrow tolerance range with low biomass, which limits the mass of metal removed per season. And most species are effective against a single contaminant class, so performance declines in precisely the mixed contamination that characterizes agricultural soils in practice. The resulting gap between ecological promise and field practicability remains substantial (Kafle et al., 2022; Mocek-Płóciniak et al., 2023; Aryal, 2024).

These constraints have prompted a new approach to remediation: instead of only designing phytoremediation strategies, researchers are focusing on the microbiome of the rhizosphere as an adaptive feature of the phytoremediation continuum. The rhizosphere is the small region of soil that is influenced by root exudates, O_2_ gradients, and rhizodeposition; it contains microbial communities whose collective catalytic and sequestering abilities far surpass those of the host plants. Root exudates (which are made up of organic acids, sugars, amino acids, and secondary metabolites) can recruit specific microbial groups to facilitate the biotransformation of pollutants, modulate heavy metal bioavailability, and mitigate phytotoxic stress (Fan et al., 2022; Martins et al., 2026; Selim et al., 2026).

### 1.1 Scope, literature selection and organizing framework

The present mini-review was based on a structured literature search of PubMed, Scopus and Web of Science complemented by backward and forward citation tracking. The last search was performed in June 2026. The review highlights sustainable rhizosphere-based remediation of agricultural soil contaminants in terms of quantifiable risk reduction, maintenance of soil productivity, avoidance of contaminant transfer, and ecological and economic cost considerations. Studies were included if they investigated microbial or plant-associated therapies such as PGPR, AMF, synthetic microbial consortia and biochar-mediated enrichment and published performance outcomes or conceptual frameworks. The evaluation has 60 sources published between 1995 and 2026, 46 (77%) of which have been published since 2020. Omics, synthetic biology, CRISPR, machine learning and digital twins are viewed as design tools, not independent remedial categories.

## 2 Mechanisms of rhizosphere engineering for contaminant remediation

Rhizosphere engineering uses the capacity of roots to shape the surrounding microbial community in order to amplify, redirect or extend the plant’s own remediation capability. Four intervention classes are treated here: PGPR bioaugmentation, AMF symbiosis, synthetic consortia and biochar-mediated enrichment (Ahkami et al., 2017; Fouad et al., 2026; Olawoyin et al., 2026).

Degradation is breakdown of the contaminant’s molecular structure, and only its endpoint, mineralization to inorganic products, removes the contaminant from the system. Transformation alters the molecule without mineralizing it, and can yield products more mobile or more toxic than the parent compound. Immobilization fixes the contaminant in place by sorption, precipitation or complexation, reducing transfer to plants and water while leaving the total amount in the soil unchanged. Reduced bioavailability lowers the fraction accessible to organisms, which may reflect genuine immobilization or merely a change in the extraction chemistry used to measure it. The distinction is sharpest for metals and metalloids, which cannot be degraded at all: for these, every microbial intervention necessarily operates through immobilization, altered bioavailability or plant uptake, and none destroys the contaminant, so immobilization should never be reported as removal. Table 1 summarizes rhizosphere microbiome-based interventions for remediating contaminated agricultural soils, highlighting the target contaminants, intervention strategies, remediation outcomes, strength of available evidence, and key research gaps.

**TABLE 1:** Rhizosphere microbiome interventions for sustainable contaminant remediation in agricultural soils.

Contaminant	Microbiome intervention	Outcome type	Evidence (scale; key result)	Key research gap	References
Cadmium	Combined PGPB inoculants (SaMR10 + SaMR12), *Sedum alfredii*	Phytoextraction (removal)	Field; straw Cd extraction rate +156% over uninoculated control	No multi-season or multi-site replication	Wang et al., 2022
Cadmium	AMF (*Glomus intraradices*) with wheat-straw biochar, maize	Immobilization; reduced bioavailability	Mesocosm; tissue Cd −50% to −76%; biomass +79%	Not replicated in the field; total soil Cd unchanged	Liu et al., 2018
Cd, Cu, Zn	Plant growth-promoting yeast *Cryptococcus* sp. NSE1, *Sedum plumbizincicola*	Phytoextraction; reduced bioavailability	Field; extractable Cd −29% to −39%; biomass +46%	Single site; persistence beyond one season not reported	Liu et al., 2016
Cd, Pb	Single wheat-straw biochar amendment, rice paddy	Immobilization	Field, 3 consecutive years; extractable Cd and Pb reduced throughout	Metal is not removed; long-term reversibility unknown	Bian et al., 2014
Cd, Pb	Sepiolite and biochar amendment, maize	Immobilization	Field; maize grain Cd −24.6% to −47.5%	Grain Pb unaffected; mechanism inferred, not measured	Zhan et al., 2019
Chlorpyrifos	Defined bacterial consortium ECO-M, bioaugmentation	Degradation to metabolites	Soil microcosm; 94.3% loss (100% in liquid culture)	No field trial; mineralization not quantified	Uniyal et al., 2021
Atrazine	Bioaugmentation with single isolates and *Paenarthrobacter* sp. W11	Transformation; partial mineralization	Soil incubation; mineralization 10% to >40% between isolates; isotope-derived degradation 29%–52%	Substantial non-extractable residue persists; no field data	Chen et al., 2021; Gallego et al., 2024
Petroleum hydrocarbons, PAHs	Five-strain hydrocarbonoclastic consortium with plant and NPK	Degradation	Field at two sites; total petroleum hydrocarbons −67% at 120 d	Mineralization not separated from sequestration	Bhuyan et al., 2023
Petroleum refinery sludge	PGPR and PAH-degrader consortium, with and without pasture plants	Degradation	Field, *in situ*; hydrocarbons declined by 7 months	Percentage removal not reported	Pizarro-Tobias et al., 2015
Microplastics (PP, PVC, PHA)	Soil bacterial isolates; organic fertilizer-stimulated biofilm	Surface transformation only	Axenic liquid culture; 7.3%–17.3% particle weight loss in 40 d. Soil incubation: weak weight loss with surface oxidation	No carbon-resolved mineralization in planted soil; no rhizosphere demonstration	Habib et al., 2020; Zhang et al., 2023
Multi-contaminant soils	*In situ* CRISPR community editing (phage-mediated or conjugative)	Targeted functional reprogramming	–	Delivery, coverage, persistence and biosafety all unresolved	Wei and Li, 2023; Shan et al., 2026

### 2.1 Plant growth-promoting rhizobacteria (PGPR)

Plant growth-promoting rhizobacteria are the predominant rhizosphere-modifying agents, and they act through two mechanistically distinct routes: catabolic transformation of organic contaminants, and modification of metal speciation and bioavailability. The catabolic route is substrate-specific rather than general. Organophosphate pesticides are hydrolysed at the phosphotriester linkage by organophosphate hydrolase (phosphotriesterase, encoded by opd and related genes), which cleaves the P-O bond to release a dialkyl phosphate and the corresponding leaving group; this single hydrolytic step accounts for most of the measured detoxification and underlies nearly all engineered organophosphate degraders (Singh and Walker, 2006). Halogenated compounds instead require reductive or hydrolytic dehalogenases, which remove halogen substituents and are mechanistically the inverse of halogenases, which install them. Polycyclic aromatic hydrocarbons are attacked by ring-hydroxylating dioxygenases, which incorporate both atoms of molecular oxygen to give a cis-dihydrodiol that is dehydrogenated to a catechol and subsequently opened by ortho- or meta-cleavage dioxygenases. These enzyme classes are not interchangeable, and a consortium assembled against one contaminant class will not act on another unless the corresponding pathway is present.

The second route alters metal speciation, and here the intended remediation objective determines whether a given bacterial activity is beneficial, because immobilization and extraction are opposing aims. Siderophores are high-affinity Fe(III) chelators of hydroxamate, catecholate or carboxylate type that also complex Cd, Pb, Zn and other metals with lower affinity. Their net effect on metal fate is bidirectional: soluble metal-siderophore complexes raise the phytoavailable pool and favor phytoextraction, whereas complexes that sorb to mineral surfaces or precipitate lower it and favor phytostabilization. Reporting siderophore production without stating which of these outcomes was measured therefore conveys little. Phosphate-solubilizing bacteria release orthophosphate by secreting gluconic and other organic acids and by lowering rhizosphere pH (Sharma et al., 2013). The consequence for metals is element-specific: released phosphate precipitates Pb as pyromorphite-type minerals and so immobilizes it, whereas phosphate competes with arsenate for the same sorption sites on Fe and Al oxides and can therefore mobilize As rather than immobilize it. Phosphate solubilization is consequently a stabilization strategy for Pb and a potential liability in As-contaminated soil, and the two should not be reported as one beneficial trait. Separately, PGPR promote growth independently of contaminant chemistry through indole-3-acetic acid and cytokinin production, which stimulates lateral root development, and through 1-aminocyclopropane-1-carboxylic acid (ACC) deaminase, which lowers stress ethylene and maintains root elongation under contaminant stress (Glick, 2012). Because this enlarges the biomass over which a given metal burden is distributed, it increases total extraction only where tissue concentration is sustained, and otherwise merely dilutes it.

### 2.2 Arbuscular mycorrhizal fungi (AMF) symbioses

Arbuscular mycorrhizal fungi extend the absorptive interface of the root through extraradical hyphae, and their contribution to remediation has to be stated against a specified objective, because immobilization and extraction pursue opposite ends. Immobilization, or phytostabilization, lowers the bioavailable metal pool and restricts transfer to shoots while leaving the total soil metal burden largely unchanged; phytoextraction seeks the reverse, maximizing translocation into harvestable tissue so that metal is physically removed from the site. AMF contribute principally to the first. Metal cations are bound by chitin and melanin components of the hyphal wall and are complexed by glomalin-related soil protein (GRSP), a glycoprotein pool of fungal origin. GRSP carries a high density of acid functional groups and binds metals mainly through deprotonated carboxyl and sulfur-containing sites, forming stable species that partition into macro-aggregates and reduce phyto-availability under field conditions (Chen et al., 2022). GRSP is also positively correlated with, and contributes measurably to, the soil organic carbon pool (Cai et al., 2023). For organic contaminants the AMF contribution is indirect. AMF possess no ligninolytic or ring-cleaving catabolic enzymes and do not themselves mineralize hydrocarbons. Their effect arises in the hyphosphere, where hyphal exudates and fungal carbon flow sustain hydrocarbon-degrading bacterial populations, so that dissipation reflects a three-component interaction between root, fungus and degrader community rather than fungal catabolism (Rajtor and Piotrowska-Seget, 2016).

### 2.3 Synthetic microbial consortia

In several experimental systems, synthetic microbial consortia assembled from taxonomically diverse strains with complementary functions outperformed single-strain inoculants for bioremediation (Wang et al., 2022; Sadvakasova et al., 2026). When designing a microbial consortium, the principle of metabolic division of labor is employed. In this principle, different strains of the consortium conduct different steps in the transformation of a contaminant. For example, some strains may carry out dehalogenation, some may perform metal reduction, or some may cleave aromatic rings. Some strains may perform growth supportive roles such as nitrogen fixation. Cross-feeding should not be treated as something to be minimized. Obligate metabolic interdependence, in which one member supplies a metabolite that another requires, is frequently engineered deliberately, because it stabilizes co-existence, enforces retention of every member and limits the escape of individual strains from the designed community. A workable consortium design therefore maximizes complementarity and interdependence while minimizing niche overlap, and the distinction between these two is the practical content of the division-of-labor principle.

### 2.4 Biochar-mediated microbiome enrichment

Biochar engineers the rhizosphere indirectly, restructuring the soil microbiome in favor of contaminant-degrading guilds (Ngaba et al., 2024; Yang et al., 2025; Li et al., 2026). Its charged, porous and persistent carbon matrix acts in three ways: it adsorbs contaminants, lowering the bioavailable concentration to sub-inhibitory levels at which microbial growth resumes; it provides habitat and refugia for slow-growing functional taxa otherwise excluded by competition; and it buffers pH and water availability against contamination-driven shifts. Figure 1 summarizes the four intervention classes and the remediation outcomes they produce.

**FIGURE 1:**
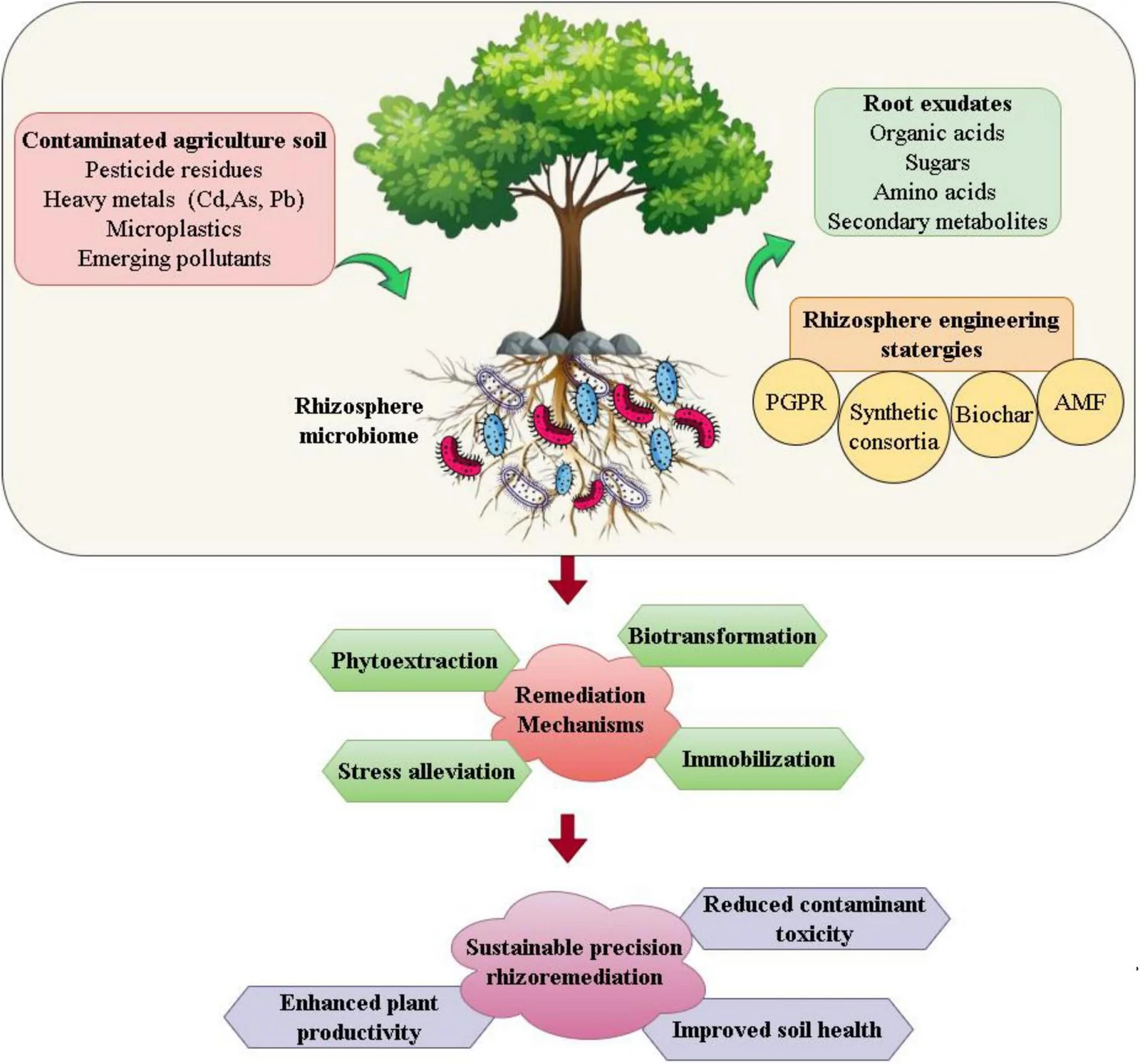
Mechanisms of rhizosphere engineering for sustainable remediation. Schematic overview of PGPR, AMF, synthetic consortia, and biochar-mediated pathways for contaminant biotransformation, immobilization, and plant-mediated extraction across organic and inorganic pollutant classes.

### 2.5 Microplastics: separating interception, transformation and mineralization

Microplastics are treated separately because the processes grouped under the heading of microplastic remediation differ in kind rather than in degree, and only one of them removes polymer from the system.

Physical interception is the obstruction of particle transport by roots, root hairs and mucilage, which concentrates particles at the rhizoplane. Adsorption is their retention on root surfaces and on soil aggregates through electrostatic and hydrophobic interaction. Neither alters polymer chemistry. Both relocate particles, and both can reduce the fraction recovered from bulk soil without reducing the quantity present, so a removal efficiency calculated from a declining bulk-soil particle count may record redistribution rather than loss.

Aging and fragmentation are driven by ultraviolet exposure, mechanical abrasion and oxidation. They introduce carbon-oxygen functional groups, raise the O/C ratio, embrittle the polymer and break particles into smaller microplastics and nanoplastics. This is a change in physical state rather than a reduction in polymer mass, and because it increases particle number and specific surface area while releasing sorbed additives it can raise rather than lower hazard (Renault et al., 2024). Surface oxidation detected by infrared spectroscopy is therefore evidence of weathering, not of degradation.

Microbial transformation is polymer-specific. Polyesters such as polyethylene terephthalate, polylactic acid, polyhydroxyalkanoates and polybutylene adipate terephthalate carry hydrolysable ester bonds and are susceptible to cutinase- and esterase-type enzymes that depolymerise them to oligomers and monomers. Polyolefins such as polyethylene and polypropylene have a chemically inert carbon-carbon backbone with no hydrolysable linkage; their attack requires oxidative initiation and proceeds orders of magnitude more slowly. Reporting both classes under a single heading of microplastic biodegradation obscures a difference of several orders of magnitude in rate.

Mineralization, the complete conversion of polymer carbon to carbon dioxide and microbial biomass, is the only one of these processes that constitutes removal, and it is the one least often demonstrated. It requires carbon-resolved evidence such as CO2 evolution or 13C or 14C labeling. Weight loss, infrared carbonyl indices and disappearance from a sieved size fraction do not establish it, because each is equally consistent with fragmentation into particles below the detection limit, and complete mineralization of environmental plastics is in fact rarely observed (Renault et al., 2024). Microplastics also act as contaminant carriers. Their hydrophobic surfaces sorb pesticides, polycyclic aromatic hydrocarbons and metals, and the particles themselves leach plasticisers, stabilizers and flame retardants (Rai et al., 2023; Zhai et al., 2024; Kundu, 2026). The remediation objective is therefore not single-valued: removing particles from the rhizosphere does not remove the sorbed or leached burden, and degrading them may release it.

## 3 Microbiome-guided design tools

To develop engineered solutions that include the plant-microbe interactions at the level of the remediation mechanism, an array of tools from molecular, computational, to synthetic biology is required. Three integrated tools of synthetic biology, CRISPR-based community editing, and omics-based strain selection allow the design of tailored rhizosphere communities focused on specific contaminants in particular soil.

### 3.1 Omics-driven strain selection

The culture-independent multi-omics approaches have greatly improved our ability to pinpoint particular rhizosphere microorganisms that possess important degradative or stabilization functions. With metagenomics, contaminated soil microbiomes can be surveyed for the functional genes that provide the capability to degrade xenobiotic compounds, and, with sequence homology search, can be utilized to identify degradative gene clusters (opd, atzA, atzB, atzC, czc) across different taxa. Metatranscriptomics provides the capability to survey expressed genes in contaminated soils (Handelsman, 2004; Rincon-Florez et al., 2013; Rajguru et al., 2024).

These establish genetic potential rather than activity, and the distinction is consequential. Detection of opd, atzA, atzB, atzC or czc establishes that a sequence related to a known determinant is present in the community. It does not establish that the gene is expressed, that its host is viable, that the remaining steps of the pathway are present in the same organism or community, or that the reaction proceeds at a rate relevant to field remediation. Partial pathways are common, and they can yield transformation products that are more mobile or more toxic than the parent compound. Gene presence is therefore a screening signal indicating where to look, and a claim of degradative capacity requires corroboration by expression data, by metabolite or isotope evidence of flux, or by measured loss of the parent compound. Root exudate metabolomics shows that contaminant stress alters exudate composition in ways that recruit different degrader taxa, which supports the design of exudate-mimicking soil amendments. Machine learning applied to multi-omics data has been proposed to predict synergistic consortium composition and functional stability under field stress, and would become necessary as consortium complexity exceeds what empirical screening can cover (Salem et al., 2022; Ali and Glick, 2024; Yang et al., 2025; Okafor et al., 2026).

### 3.2 Synthetic biology (SynBio) for rhizosphere engineering

Synthetic biology extends design beyond strain selection. Degradation pathways have been transferred into rhizosphere-competent hosts to widen substrate range, and contaminant-responsive biosensors have been used to couple pathway expression to contaminant concentration, in principle matching catabolic effort to need (Aminian-Dehkordi et al., 2023; Maqsood et al., 2024; Adhikary et al., 2026).

### 3.3 CRISPR-based functional microbiome design

CRISPR-Cas tools are proposed to extend microbiome engineering from single-strain modification to community-level functional editing. In cultured strains the component technology is established: CRISPR interference (CRISPRi) represses transcription reversibly and in a dose-dependent manner, which in principle allows the output of a degradative pathway to be balanced against its cost to the host cell, and Cas12a-based systems extend this to the simultaneous regulation of several genes (Fleck and Grundner, 2021; Ali et al., 2026; Shan et al., 2026; Singh et al., 2026). Extending that control from cultured strains to resident soil communities is, however, a proposal rather than a demonstrated capability. A distinction should be drawn that this literature often leaves implicit. CRISPRi as demonstrated is a method for regulating gene expression in cultured, genetically tractable and usually axenic strains, where the construct is delivered by transformation and maintained under selection. Community-level editing in soil is a different proposition, requiring delivery to uncultured and taxonomically diverse recipients without selection, coverage of a sufficient fraction of the target population to alter community function, and persistence of the effect against dilution by growth and loss of the construct.

Omics-based survey is the most mature: metagenomic and metatranscriptomic profiling of contaminated soils is routine, and consortia built from isolates screened for the corresponding catabolic traits have been validated in the field (Bhuyan et al., 2023). Synthetic biology constructs and CRISPRi are demonstrated in cultured, genetically tractable strains, and in those systems only. AI-guided consortium design, microbiome-informed digital twins and *in situ* editing of resident soil communities have been proposed and modeled but not demonstrated in any soil system.

### 3.4 Stratifying the evidence base

The evidence supporting microbiome-guided rhizoremediation is distributed unevenly across both contaminant classes and experimental scales, and conflating these tiers has obscured how much of the field remains unvalidated. Established, field-validated evidence is at present confined to two domains. The first is metal immobilization and metal phytoextraction. Combined PGPB inoculants raised straw Cd extraction rates by 156% under field application (Wang et al., 2022); a plant growth-promoting yeast increased hyperaccumulator biomass by 46% and reduced HCl-extractable and CaCl2-extractable soil Cd by 39.2% and 29.5% in field experiments (Liu et al., 2016); a single biochar amendment reduced extractable Cd and Pb continuously across three consecutive paddy seasons (Bian et al., 2014); and sepiolite and biochar reduced maize grain Cd by 24.6%–47.5% on mine-adjacent farmland (Zhan et al., 2019). The second domain is hydrocarbon rhizoremediation, where a five-strain hydrocarbonoclastic consortium combined with *Azadirachta indica* and NPK biostimulation degraded up to 67% of total petroleum hydrocarbons over 120 d at two contaminated field sites (Bhuyan et al., 2023), and an artificial consortium of PGPR and PAH degraders reduced petroleum hydrocarbons in an *in situ* semiarid field trial (Pizarro-Tobias et al., 2015). Both domains rely on bioaugmentation with cultured isolates or on bulk amendment; neither depends on genome-edited organisms.

Arbuscular mycorrhizal fungi inoculation combined with biochar reduced maize tissue Cd by 50%–76% in mesocosm (Liu et al., 2018), and the ECO-M bacterial consortium removed 94.3% of chlorpyrifos from contaminated soil in microcosm (Uniyal et al., 2021). The distance between such figures and field performance is best illustrated by atrazine, where isotope-resolved work showed mineralization varying from 10% to above 40% between isolates, with a substantial non-extractable residue persisting in every treatment (Gallego et al., 2024). Seed-delivered SynComs can dominate the seedling microbiota, contributing on average 80% of its membership and displacing native seed and soil taxa (Arnault et al., 2024), which establishes that deliberate community installation is achievable. Yet in natural low-fertility soil, commercial PGPR failed to colonize at all and increased niche overlap by 59.2%–62.4%, intensifying competition, whereas a native SynCom raised the maize root: shoot ratio by 78%–121% against 23%–86% for the commercial product (Jiang et al., 2023).

Speculative and conceptual claims form the third tier and should be labeled as such. Microplastic rhizoremediation is the clearest case, and plant-based strategies for it have recently been reviewed (Nair et al., 2026). The most frequently cited degradation figures, 17.3% and 7.3% particle weight loss over 40 d, were obtained in axenic mineral salts medium without soil, plant or rhizosphere (Habib et al., 2020), while a 240-d soil incubation detected only weak weight loss accompanied by surface oxidation, without quantifying mineralization (Zhang et al., 2023). Surface alteration is not removal, and no rhizosphere-mediated microplastic remediation efficiency has yet been demonstrated in soil. *In situ* CRISPR community editing is more speculative still (Wei and Li, 2023; Shan et al., 2026). The same applies to microbiome-informed digital twins and AI-guided consortium design, which remain computational proposals without reported field validation.

## 4 Field implementation challenges

### 4.1 Formulation, carrier selection and shelf life

Bringing successful laboratory rhizosphere engineering examples to robust, large-scale field remediation presents a number of challenges that have not been thoroughly resolved, especially concerning practicality and regulations. Two major challenges remain formulation and delivery. The currently available carrier systems for PGPR and consortia (peat, alginate beads, wettable powders) suffer transport and retention problems under field relevant stress (temperature, moisture, UV, etc.,) and have a shelf life that is less than the 6–12 months necessary for successful commercialization. While there is active research around encapsulation technologies (e.g., clay minerals, polymer nano-capsules, biofilm hydrogel matrices), field scale testing has yet to be achieved (Mahmud et al., 2021; Adhikary et al., 2026).

The figure of 6–12 months as an indicative commercial expectation rather than a measured property of all formulations: shelf life is strain-, carrier- and storage-specific, ranging from weeks for unformulated liquid suspensions to substantially longer for lyophilized or immobilized preparations held refrigerated, and any specific figure should be reported with the formulation and storage regime under which it was measured. Carrier choice governs this directly, because the carrier supplies the physical and nutritional matrix that sustains cell viability between manufacture and application, and the available materials differ widely in water-holding capacity, buffering, sterilisability and cost. Cell immobilization and the use of cell-free filtrates are among the strategies developed to extend viability without compromising activity (Khan et al., 2023).

### 4.2 Spatial heterogeneity and variable-rate application

Within agricultural ecosystems, the contamination of field soil is highly variable and results in the misalignment between the optimized laboratory conditions of inoculants and the field conditions. For instance, strains of bacterial inoculants that are selected to thrive at the maximum contaminated field concentration may not perform adequately in the majority of the contaminated field, where the contaminant concentration is significantly lower (Trabelsi and Mhamdi, 2013; Li et al., 2026). One prospective solution to this challenge is the application of variable rate inoculant systems aligned to contamination mapping, which is dependent on the development of affordable contaminant sensing technologies that can be applied at field scale.

### 4.3 Establishment and persistence under field conditions

Establishment is the step at which most inoculant programmes fail, and failure is frequently silent because it is not measured. Commercial plant growth-promoting rhizobacteria applied to natural low-fertility soil did not colonize at all, while a native synthetic community assembled from the same soils established and conferred a growth benefit, indicating that provenance governs establishment more strongly than designed function (Jiang et al., 2023). Delivery route also matters: seed-applied synthetic communities can dominate the seedling microbiota, contributing on average 80% of its membership, because they occupy the niche before the resident community assembles (Arnault et al., 2024). Against this, introduced populations typically decline over weeks to months as resident competition and predation reassert themselves, and the resulting perturbation of the native community may outlast the inoculant itself (Trabelsi and Mhamdi, 2013).

### 4.4 Cost, land value and the economic case

Economic viability constrains deployment at least as strongly as biology, and it is rarely addressed in the rhizosphere engineering literature. Techno-economic analysis of phytoremediation indicates that the decisive variables are the time required for treatment and the opportunity cost of the land occupied during it: the approach is most competitive in remote regions of low land value, where extended treatment periods are tolerable, and is correspondingly difficult to justify where land values are high and cannot be recovered over a short horizon (Wang and Aghajani Delavar, 2023). Microbiome-guided intervention is attractive precisely because it shortens that timeline, so the economic case for it should be made in those terms, as a reduction in years of land occupancy, rather than as a removal percentage.

### 4.5 Verification of field performance

Field verification raises difficulties that laboratory work does not. Contaminant concentrations in field soil vary over meters, so an apparent reduction may reflect sampling position rather than treatment, and paired pre-treatment and post-treatment sampling at fixed georeferenced points is necessary to separate the two. Natural attenuation proceeds in untreated soil, so an unamended control plot is required before any reduction can be attributed to the intervention. Dilution by tillage, erosion and leaching can lower a measured concentration without any contaminant being degraded, which is why mass balance over a defined soil depth is more informative than concentration alone.

## 5 Biosafety, ecological risk and regulatory considerations

Bioaugmentation with cultured native isolates, and bulk amendments such as biochar, raise questions of ecological disturbance but not of genetic novelty; these are the approaches for which field validation exists. Engineered strains, consortia carrying heterologous catabolic pathways, and *in situ* community editing by phage-mediated or conjugative delivery raise both, and these are the approaches for which no field data exist. Biosafety analysis is therefore not an appendix to this review but the principal obstacle standing between its two evidence tiers.

### 5.1 Horizontal gene transfer

The rhizosphere is among the more permissive environments for horizontal gene transfer, because high cell density, exudate-derived carbon and surface contact all favor conjugation. Direct measurement in fabricated rhizosphere ecosystems has shown plasmid transfer between *Pseudomonas putida* donors and recipients, and onward to a different root-colonizing genus, at frequencies that vary with plant developmental stage, root exudate composition and quantity, donor cell density and environmental stress (Priya et al., 2024). Soil bacteria additionally carry large integrative and conjugative elements that move readily between genomes and can exceed 800 kb, so mobility is not restricted to small plasmids (Colombi et al., 2021). This matters particularly for *in situ* editing strategies, because conjugative delivery deliberately exploits the same machinery: a delivery system optimized for transfer efficiency is, by construction, optimized for escape, and the two properties cannot be independently tuned. Any proposal to reprogram resident communities by conjugation should therefore report transfer frequencies to non-target taxa alongside transfer to intended recipients.

### 5.2 Persistence, dispersal and ecological containment

Containment strategies for engineered microorganisms rely mainly on auxotrophy and on kill switches, and the evidence suggests that neither should be equated with removal. In a model engineered organism carrying a CRISPR-Cas9 kill switch, escape rates measured by abundance of the targeted genes were several orders of magnitude higher than escape rates measured by colony-forming units, because the switch arrests colony growth while leaving a large fraction of target genes intact and detectable; the DNA remained inside intact membranes and resistant to DNase for a period after containment (Hartig et al., 2026). Two consequences follow. First, biological containment suppresses proliferation without eliminating the genetic construct, so recombinant DNA persists as a substrate for transfer after the host is no longer viable. Second, culture-based monitoring systematically understates persistence, and a containment claim supported only by plate counts is not supported.

### 5.3 Non-target effects and microbiome perturbation

Perturbation of the resident community is not peculiar to engineered organisms; it accompanies inoculation generally, and its magnitude and reversibility, rather than its occurrence (Trabelsi and Mhamdi, 2013). The quantitative evidence is sobering even for unmodified commercial products: in low-fertility soil, commercial PGPR that failed to establish nonetheless narrowed niche breadth and raised niche overlap among resident taxa by 59.2%–62.4%, intensifying competition (Jiang et al., 2023). An inoculant can therefore disturb community structure without delivering the function it was applied for, which is the least favorable of the possible outcomes.

### 5.4 Resistance evolution

Any engineered function imposing a fitness cost selects for its own loss, and any system that kills selects for escape from killing. Kill switches select for inactivating mutations in the switch itself, which is the mechanism underlying the escape frequencies noted above. CRISPR-based community editing faces an additional and specific obstacle, because anti-CRISPR proteins are widespread among phages and mobile elements and provide resident bacteria with a pre-existing route to neutralize introduced CRISPR systems (Shan et al., 2026). Selection will also act on the delivery vehicle, through restriction-modification systems, surface receptor loss and abortive infection. Engineered constructs that retain antibiotic resistance markers, still common in laboratory strains, add a further and avoidable hazard in an agricultural soil. Durability under field selection should therefore be reported as an experimental result over seasons, not assumed from laboratory-scale stability.

### 5.5 Regulatory pathways and monitoring requirements

Regulation of environmental release of genetically modified microorganisms remains fragmented: responsibility rests with individual national frameworks and there is no coordinated international policy governing release or post-release surveillance, a gap that has prompted calls for a staged, decision-based biosafety workflow combining pre-release risk assessment with continuous post-release monitoring under the precautionary principle (Lerner et al., 2026). For rhizosphere engineering specifically, three monitoring requirements follow from the evidence above. Monitoring must be molecular rather than culture-based, and must track construct abundance and not only viable cell counts, since the two diverge by orders of magnitude. And it must include transfer to non-target taxa as a measured endpoint wherever mobilizable elements are used.

## 6 Evidence gaps and limitations

### 6.1 Gaps in the primary evidence base

Five gaps recur across the contaminant classes surveyed. First, no microbiome-guided intervention has been evaluated in multi-season, multi-site field trials under a common reporting standard, so between-study variation cannot be separated from site effects. Second, framed on co-contaminated soils, interaction effects between metals, pesticides and plastics on degrader performance remain essentially unquantified. Third, inoculant application rates are seldom varied systematically, so there is no dose-response basis for choosing a field rate. Fourth, the trade-off between remediation performance and crop yield, although frequently invoked as a motivation, is rarely measured in the same experiment as the remediation endpoint; where yield is reported it is usually biomass under controlled conditions rather than marketable yield under field competition. Fifth, inoculant survival beyond a single season is rarely reported at all, so the question of whether reapplication is required each year is unresolved for every approach surveyed.

### 6.2 Methodological limitations in the reported literature

Several limitations are properties of how the literature is reported rather than of the underlying biology. Removal is routinely conflated with mineralization, transformation and sequestration, which are different fates with different risk implications. Efficiency percentages are not comparable between studies because contaminant loading, soil type, plant system, exposure duration and analytical endpoint all differ, and a minimum reporting set covering these five variables would do more for cumulative progress than additional single-site demonstrations. Culture-based enumeration understates persistence relative to molecular measurement. Baselines and time series are frequently replaced by single post-treatment comparisons, which cannot distinguish treatment effect from pre-existing difference. Finally, unsuccessful inoculations are unlikely to be published at the rate they occur, so the central tendency of the reported literature probably overstates field performance; the colonization failure documented by Jiang et al. (2023) is informative precisely because such results are seldom reported.

## 7 Future perspectives

Three developments are repeatedly identified as the next step, and all three are at present proposals rather than tools. Microbiome-informed digital twins, parameterized with site-specific omics, soil and contaminant data, could in principle pre-optimize consortium composition and application timing before deployment; a systematic review of digital twins applied to soil quality nevertheless found no published application within a predictive-control context (Silva et al., 2023; Bradbury et al., 2025; Kumar et al., 2026). AI-guided consortium design is constrained less by model architecture than by the volume of standardized field performance data available for training, and which open data practice would address. *In situ* editing by phage-mediated or conjugative CRISPR delivery would bypass the establishment barrier altogether, and has been demonstrated in gut communities but not in soil (Stindt and McClean, 2024; Ronda et al., 2025; Narayanan, 2026).

## 8 Conclusion

Microbiome-guided rhizosphere engineering is a maturing rather than a mature field, and its readiness differs sharply by approach. Bioaugmentation with cultured isolates and bulk amendment have been validated in field trials for metal immobilization, metal phytoextraction and hydrocarbon dissipation, and for these a practical case can be made now. The engineered and computational approaches that dominate recent discussion, including consortia carrying heterologous pathways, *in situ* CRISPR community editing, digital twins and AI-guided design, remain at proof of concept and have no field validation; for these the appropriate claim is promise rather than demonstrated performance. Convergent advances in multi-omics-driven strain selection, synthetic biology, and CRISPR-based community editing have created a richer toolkit than was available a decade ago. Yet field performance remains constrained by ecological instability of introduced inoculants, context-dependency of plant-microbe interactions, incomplete understanding of microbiome assembly rules, and regulatory ambiguity for engineered organisms. Closing these gaps requires longitudinal, multi-site field trials with standardized reporting; deeper integration of microbiome ecology with synthetic biology; and development of digital twin and AI-guided design platforms. With these advances, precision, data-driven rhizosphere engineering could transition from a research paradigm to a deployable technology for sustaining agricultural productivity on contaminated lands.
